# Elucidating Temporally Ordered Physiological Events During Liver Regeneration via Integrated Transcriptomic-Histological Profiling

**DOI:** 10.3390/ijms27198829

**Published:** 2026-10-02

**Authors:** Takashi Nishi, Yoshinao Oki, Reiko Hagiwara, Koichiro Kano

**Affiliations:** 1 Laboratory of Cell and Tissue Biology, College of Bioresource Sciences, Nihon University, 1866 Kameino, Fujisawa 252-0880, Japan; takashi.nishi@med.toho-u.ac.jp (T.N.); reiko.hagiwara@fujita-hu.ac.jp (R.H.); kano.kouichirou@nihon-u.ac.jp (K.K.); 2 Department of Biochemistry, School of Medicine, Faculty of Medicine, Toho University, Omori-Nishi, Tokyo 143-8540, Japan; 3 Division of Gene Regulation, Oncology Innovation Center, Fujita Health University, Toyoake 470-1192, Japan

**Keywords:** liver regeneration, partial hepatectomy, microarray analysis

## Abstract

Although the liver possesses remarkable regenerative capacity, the detailed chronology spanning the pre-proliferative preparatory stage, hepatocyte proliferation, liver weight recovery, and the completion of molecular regeneration has not been fully elucidated. Using microarray-guided histological validation, we investigated the temporally ordered physiological events underlying liver regeneration following 70% partial hepatectomy (PH) in C57BL/6 mice. Our results demonstrated that PH induced multiple functional pathways, including the response to stilbenoid, steroid hormone metabolism, lipid transport, and angiogenesis. This was followed by activation of endoplasmic reticulum (ER) stress and insulin processing, accompanied by lipid accumulation, suppression of xenobiotic catabolic capacity, and altered circulating cholesterol levels. After recovery of these processes, hepatocytes and sinusoidal endothelial cells entered the proliferative phase. Notably, although liver weight had returned to preoperative levels, the transcriptome remained distinct from baseline, revealing divergence between macroscopic recovery and molecular-level convergence. At 336 h, two of three samples were assigned to the baseline Cluster 1, whereas one sample remained in Cluster 6 with a small number of altered genes. Together, these findings provide a detailed temporal framework in which metabolic and tissue remodeling during liver regeneration extends beyond macroscopic recovery.

## 1. Introduction

The liver is distinguished both by its remarkable regenerative capacity and by its central role in synthesizing, storing, metabolizing, and redistributing the three major nutrients: carbohydrates, proteins, and lipids. Following 70% surgical partial hepatectomy (PH), the remaining liver tissue undergoes rapid hypertrophy, with hepatocytes proliferating to compensate for the loss of tissue and function [1,2]. During regeneration, hepatocytes face substantial physiological demands because they must divide while maintaining essential metabolic functions, including blood protein synthesis, bile secretion, and biodegradation of noxious substances required for homeostasis [3]. Because the liver plays a central role in gluconeogenesis, rodents subjected to PH become transiently hypoglycemic [4,5]. The remaining hepatocytes also accumulate lipids transiently during this period; disrupting this adaptive response impairs hepatocyte proliferation and delays regeneration [6]. Because liver regeneration after PH is widely accepted to occur through hepatocyte hypertrophy and proliferation rather than stem cell-mediated regeneration [7,8], these metabolic perturbations have been implicated in the initiation of hepatocyte proliferation. Consistent with this concept, interventions that counteract or delay these metabolic changes—including intravenous glucose supplementation [9] or delayed onset of PH-induced hypoglycemia [10]—inhibit or postpone hepatocyte proliferation. These observations support a close temporal association between the early metabolic response and the subsequent proliferative program.

Most liver regeneration studies have focused on the proliferative phase, particularly the mechanisms that regulate hepatocyte proliferation. Relatively few studies, however, have comprehensively characterized the chronological sequence from pre-proliferative preparation to completion of regeneration. Traditionally, completion of liver regeneration has been defined by the recovery of macroscopic indicators, such as liver weight and serum biochemical parameters. Whether restoration of these macroscopic indices reflects a return to the preoperative molecular state, particularly at the transcriptomic level, remains unclear. Moreover, the frequent use of pooled samples or few biological replicates at each time point has limited characterization of the temporal dynamics of molecular recovery.

Defining the complete chronology of physiological events and molecular recovery therefore requires a comprehensive, unbiased analytical approach. Large-scale gene-expression profiling enables objective evaluation of dynamic tissue states throughout regeneration. In a pioneering rat 70% PH study, investigators used a custom cDNA microarray containing 4608 clones from a normalized, liver-enriched cDNA library to profile gene expression from immediately after surgery through 1 week post-PH. The resulting microarray profiles closely reflected the in vivo physiological changes [11]. However, this platform was restricted to a curated liver-enriched gene set rather than providing genome-wide coverage. The study also lacked comprehensive histological validation and did not evaluate complete transcriptional convergence to the preoperative baseline.

A recent mouse study similarly used microarray analysis to identify regulators of hepatocyte proliferation, but sampled tissue only at 48 and 72 h after hepatectomy [12]. To date, no study has captured the complete chronological trajectory from the initial metabolic response through to full transcriptional recovery in a 70% PH mouse model. Nor has any previous study examined the full trajectory by integrating time-course transcriptomic profiling with systematic histological validation. Although bulk transcriptomic analysis provides comprehensive molecular information, it does not provide spatial or physiological context. Specifically, it cannot establish whether transcriptional changes correspond to altered protein expression or tissue architecture in particular cell populations.

As a data descriptor, we previously published a time-course microarray dataset spanning 0–14 days after PH, with three biological replicates at each of 11 time points [13]. Here, we present the first comprehensive biological analysis of this dataset. By combining X-means clustering with Gene Ontology (GO) analysis of differentially expressed genes (DEGs), we characterized the functional pathways associated with each regenerative phase. To confirm and extend the transcriptomic findings, our strategy linked data-driven transitions in GO terms—including lipid metabolism, angiogenesis, and stress response—to chronological histological validation.

By integrating transcriptomic and histological data, we characterized a temporally ordered series of physiological events during liver regeneration. These events included characteristic fluctuations in serum high-density lipoprotein (HDL) and low-density lipoprotein (LDL) concentrations and coordinated proliferation of hepatocytes and hepatic sinusoidal endothelial cells that conventional weight- or function-based assessments do not capture. Notably, even after liver weight had recovered to preoperative levels at 96 h, the transcriptome remained distinct from the baseline, indicating a divergence between macroscopic recovery and molecular-level (transcriptional) convergence. Analysis of multiple biological replicates at each time point also revealed differences among the transcriptional profiles of the three samples at 336 h. By 336 h, most animals had achieved near-complete convergence to the preoperative transcriptome, whereas one of the three samples retained a small number of altered genes at 336 h, suggesting potentially delayed transcriptional convergence. Together, this study reframes liver regeneration not merely as cell proliferation but as a temporally ordered program of metabolic and tissue remodeling that extends beyond macroscopic recovery.

## 2. Results

### 2.1. Liver Weight Recovery Precedes Transcriptional Convergence During Regeneration

**Figure 1:**
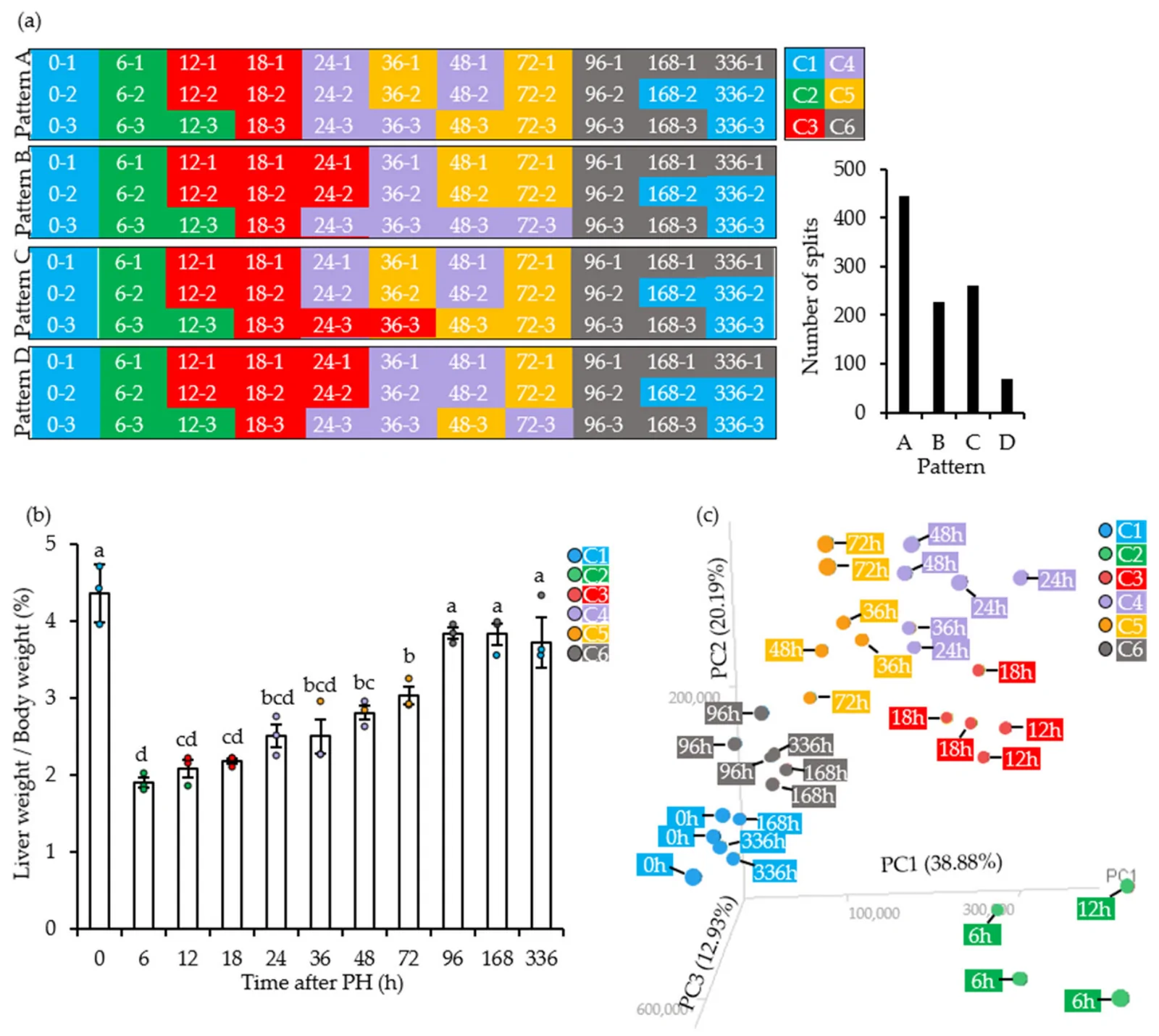
Liver gene-expression dynamics during regeneration after partial hepatectomy (PH). (**a**) X-means clustering of samples. Six clusters were identified, and the graph shows the frequency of each clustering pattern across X-means iterations. (**b**) Liver-to-body weight ratios during regeneration. The 0-h value represents the intact whole liver before PH. Data points are colored according to the X-means clusters in (**a**). Values are means ± standard error of the mean (SEM; *n* = 3). Different letters indicate significant differences between groups (*p* < 0.01, Tukey’s test). (**c**) Three-dimensional principal component analysis (3D-PCA) of the microarray data. Point labels indicate time after PH, and colors indicate the X-means clusters in (**a**). PC1, PC2, and PC3 explained 38.88%, 20.19%, and 12.93% of the variance, respectively.

We first evaluated macro-anatomical recovery across 11 time points (0–336 h post-PH) in a murine 70% PH model to characterize the physiological and molecular transitions during liver regeneration. Three independent mice were analyzed at each time point (*n* = 3 per time point). Immediately after resection, the liver-to-body weight ratio decreased significantly and then gradually recovered. By 96 h post-PH, it did not differ significantly from the preoperative baseline (0 h) (one-way ANOVA followed by Tukey’s post-hoc test, *p* < 0.01; Figure 1b).

We extracted total RNA from the same triplicate liver samples used for the anatomical analysis (*n* = 3 per time point) and performed high-precision cDNA microarray profiling to define the molecular dynamics of regeneration. Despite the tightly controlled design, individual mice may differ subtly in regenerative progression at a given time point. We therefore used unsupervised X-means clustering to classify samples by transcriptomic state rather than chronological time alone. We assessed clustering reproducibility and stability by running the X-means algorithm 1000 times; random centroid initialization in the underlying *k*-means algorithm introduced stochastic variation.

Across 1000 iterations, the samples consistently formed six distinct clusters, although the analysis yielded four closely related clustering patterns (Patterns A–D) (Figure 1a). The six transcriptomic clusters derived from the most frequently identified pattern (Pattern A) are hereafter referred to as Clusters 1–6. Pattern A occurred most frequently (444 of 1000 runs), followed by Patterns C (261 runs), B (226 runs), and D (69 runs). The four patterns differed exclusively in the assignment of samples collected 24–72 h post-PH. By contrast, the early (0–18 h) and late (96–336 h) phases were classified identically in every iteration. We therefore selected Pattern A as the most robust and representative scheme for downstream analyses. This selection did not affect the principal conclusions because Patterns B–D differed only in their assignment of intermediate-phase samples, not in the overall transcriptomic trajectory or composition of terminal Cluster 6.

We next asked whether transcriptional recovery paralleled restoration of organ weight. Superimposing Pattern A on the anatomical recovery timeline revealed a clear discrepancy between macroscopic and molecular recovery (Figure 1b). Although the liver-to-body weight ratio had returned to the preoperative baseline by day 4 (96 h), the global gene-expression profile at this stage (Cluster 5) remained clearly segregated from the preoperative transcriptome (Cluster 1, 0 h). Thus, active tissue and metabolic remodeling continued at the molecular level despite anatomical and gravimetric recovery, indicating prolonged transcriptional convergence toward the preoperative state.

We performed three-dimensional principal component analysis (3D-PCA) with PC1, PC2, and PC3 to visualize the continuous transcriptomic trajectory during liver regeneration; together, these components explained 72% of the total variance (Figure 1c). When overlaid on the 3D-PCA plot, samples assigned to the same X-means cluster grouped closely in transcriptomic space, supporting the robustness of the clustering. Clusters 3, 4, and 5 contained the 24–72 h samples responsible for variation among the four X-means patterns and occupied neighboring positions in PCA space. Their proximity reflected a highly dynamic but continuous transition through the intermediate regenerative stages.

Over time, the transcriptomic trajectory followed a distinct loop-like path. Representing the acute-phase response, Cluster 2 (6 and 12 h samples) was displaced furthest from baseline Cluster 1 (0 h), primarily along the PC1 axis. The transition from Cluster 2 to Cluster 3 (containing the 12- and 18-h samples) was characterized by substantial shifts along both the PC1 and PC2 axes. The transcriptome then progressed sequentially through Clusters 4 and 5 before entering Cluster 6, which comprised the late-phase samples and was closest to the preoperative baseline.

**Figure 2:**
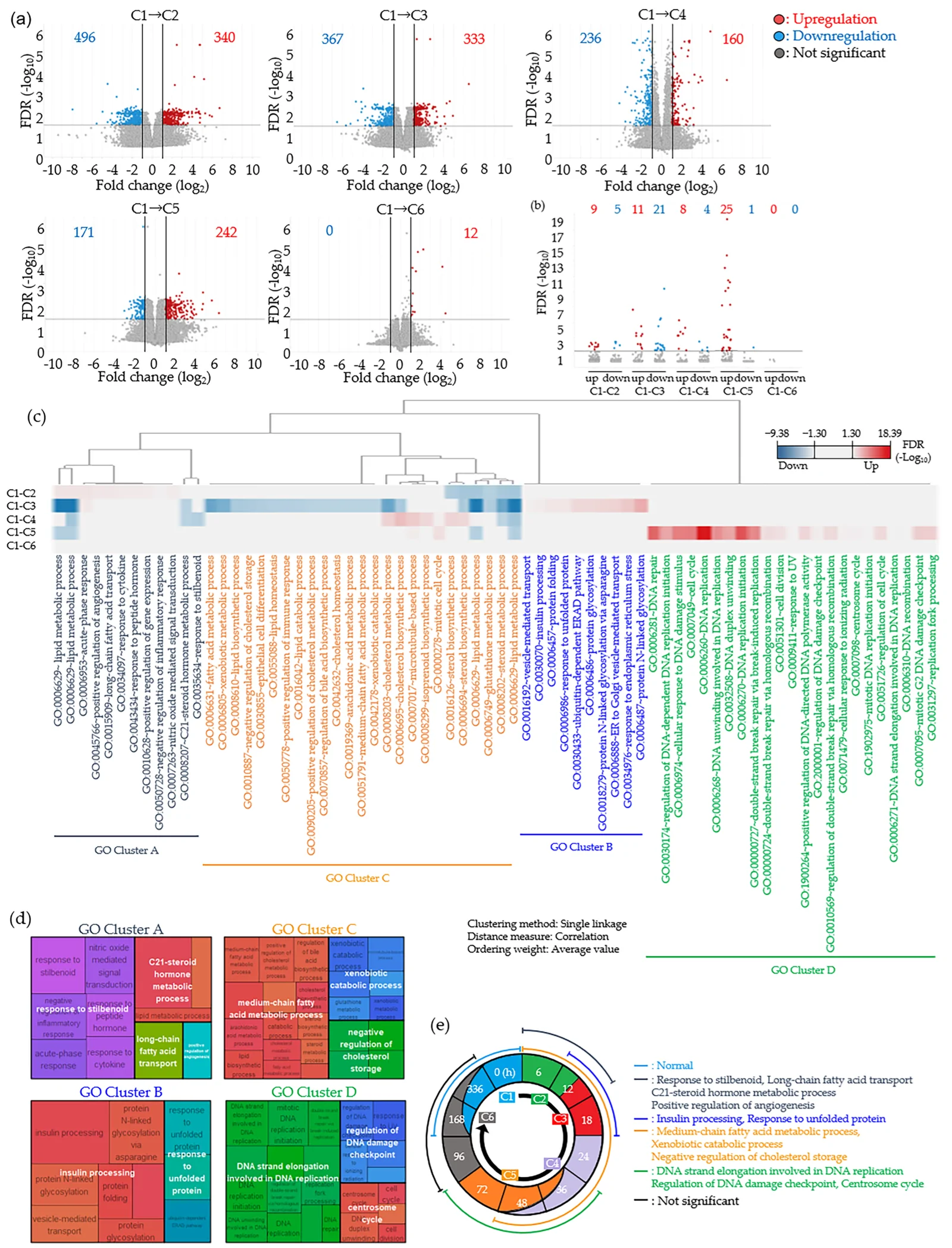
Functional analysis and Gene Ontology (GO) clustering of differentially expressed genes (DEGs). (**a**) Volcano plots comparing gene expression in the first cluster (C1) with that in each cluster. DEGs were defined as genes with greater than twofold expression changes and a false discovery rate (FDR) < 0.05 relative to C1. The x- and y-axes show fold change and FDR, respectively. Dots represent individual genes, and numbers indicate total DEGs (red, upregulation; blue, downregulation). (**b**) GO terms identified by functional enrichment analysis at FDR < 0.05. The *y*-axis shows the FDR. Dots represent individual GO terms, and numbers indicate total terms (red, upregulation; blue, downregulation). (**c**) Heat map and hierarchical clustering of enriched GO terms; colors represent FDR (red, upregulation; blue, downregulation). (**d**) REVIGO summary of each GO cluster. (**e**) Schematic overview of gene-expression changes during liver regeneration after 70% PH. The time axis shows post-resection time points, and the background color indicates the X-means cluster assigned at each time point. Curves in the outer ring show the GO clusters that changed at each time point; the legend lists the GO terms in each cluster.

At the final time point (336 h), the three biological replicates did not converge uniformly. Two of the three samples were assigned to the baseline Cluster 1, whereas the remaining sample was assigned to Cluster 6. Cluster 6 was extremely close to Cluster 1 in 3D-PCA space, but a small number of genes remained significantly differentially expressed between them (Figure 2a). Thus, the three samples did not all show transcriptional convergence to the baseline cluster by 336 h. This observation suggests that the timing of transcriptional convergence may differ among samples, although the small sample size precludes a definitive assessment of inter-individual heterogeneity.

### 2.2. Functional Annotation Reveals Sequential Transcriptional Programs During Liver Regeneration

We identified DEGs in each sample cluster relative to C1 to characterize the biological roles of the six clusters (C1–C6). The number of DEGs peaked in C2, with a total of 836 genes (496 downregulated and 340 upregulated), and remained substantial from C3 through C5. In contrast, C6 exhibited minimal transcriptional deviation, with only 12 upregulated and no downregulated DEGs (Figure 2a). This minimal deviation was consistent with the proximity of C6 to C1 in the 3D-PCA trajectory (Figure 1c) and with C6 comprising only the single 336-h replicate that had not converged to the C1 state. Functional enrichment analysis showed that downregulated GO terms were most numerous in C3, whereas upregulated GO terms peaked in C5. No GO terms were enriched in C6 (Figure 2b), indicating that the residual differences from baseline involved a few individual genes rather than a coordinated functional program.

Hierarchical clustering organized all enriched GO terms into four functional modules (GO Clusters A–D), simplifying the complex transcriptomic dataset (Figure 2c). REVIGO summarization revealed a highly ordered physiological sequence (Figure 2d,e). GO Cluster A (6–12 h) was characterized by early upregulation of the response to stilbenoid, C21-steroid hormone metabolic process, LCA transport, and angiogenesis. Induction of insulin processing and the response to unfolded proteins (endoplasmic reticulum (ER) stress) defined GO Cluster B (12–18 h). GO Cluster C (6–48 h) exhibited sustained downregulation of medium-chain fatty acid metabolism, xenobiotic catabolic processes, and negative regulation of cholesterol storage. Finally, late-phase activation of DNA strand elongation, the DNA damage checkpoint, and the centrosome cycle characterized GO Cluster D (36–72 h).

No significant GO-level functional alterations were detected beyond 96 h, indicating convergence of the major functional pathways toward the preoperative state by 168 h. In the single replicate still classified as C6 at 336 h, however, a small number of DEGs (*n* = 12) remained significantly upregulated relative to C1. Thus, despite resolution of pathway-level functional signatures across the population, complete gene-level convergence had not occurred in every individual. The resulting spatiotemporal roadmap (Figure 2e) therefore distinguishes functional (GO-level) recovery, which was largely complete by 168 h, from full transcriptional (gene-level) convergence, which remained nonuniform across the three samples at the latest time point.

### 2.3. GO Cluster A: Early Post-PH Activation of Angiogenic Signaling, Steroid/Drug Metabolism, and Lipid Uptake

**Figure 3:**
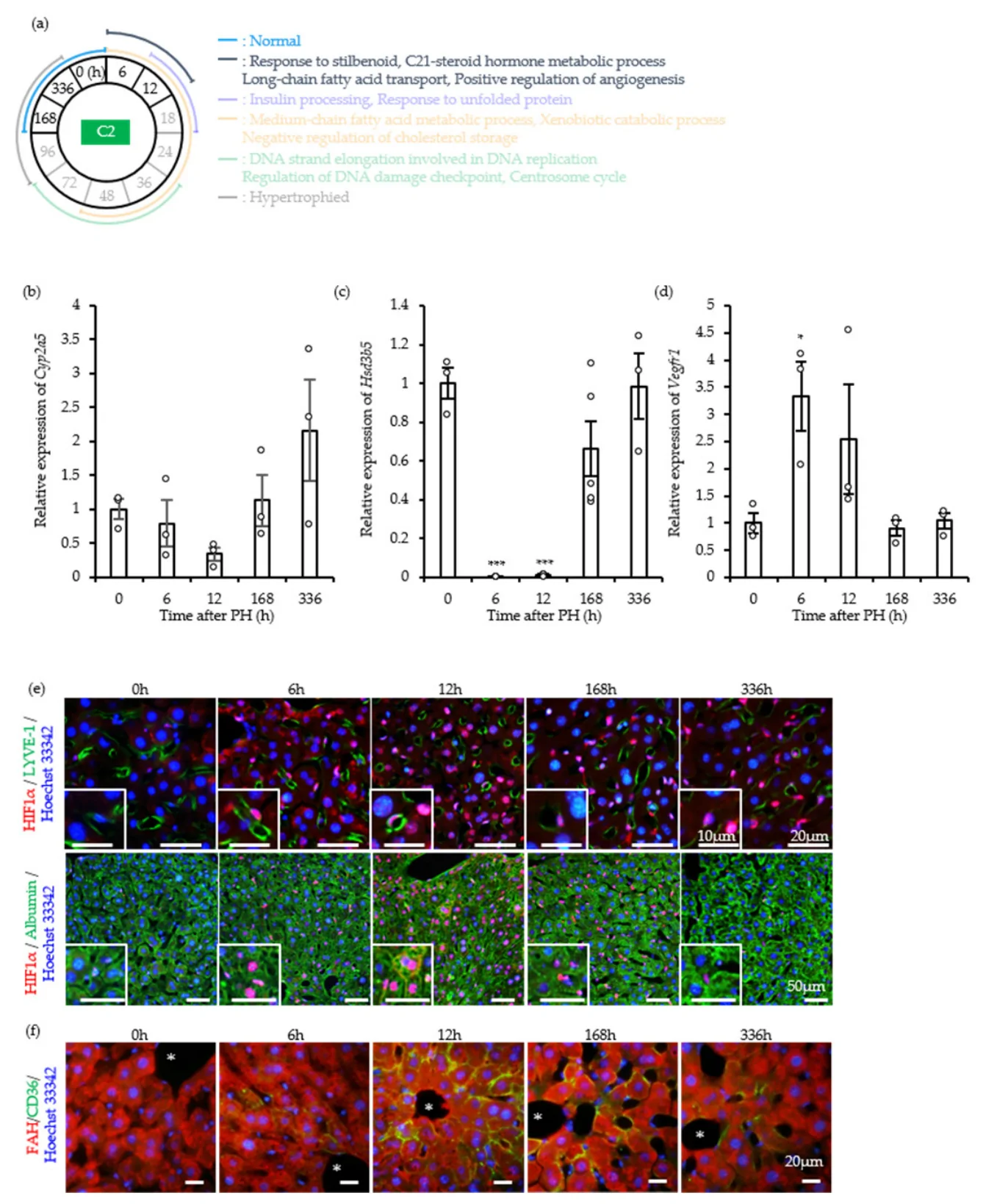
Stilbenoid response, steroid hormone metabolism, angiogenesis, and long-chain fatty acid (LCFA) transport in GO Cluster A. (**a**) Schematic overview of the post-PH time points and biological events validated in vivo. The circle center identifies the cluster analyzed, and highlighted text identifies the post-resection time points and corresponding GO terms examined. (**b**–**d**) Expression of *Cyp2a5* (**b**), *Hsd3b5* (**c**), and *Vegfr1* (**d**). Values are means ± SEM, with individual data points shown (*n* = 4 or 5 mice per time point, empty circles). * *p* < 0.05, *** *p* < 0.001 vs. 0 h (one-way ANOVA followed by Dunnett’s post-hoc test). (**e**) Immunofluorescence staining of HIF-1α (red), LYVE-1 or Albumin (green), and Hoechst 33342 (blue). Scale bars, 50, 20, or 10 μm. (**f**) Immunofluorescence staining of CD36 (green), FAH (red), and Hoechst 33342 (blue). Scale bar, 20 μm.

We performed targeted validation at 6 and 12 h post-PH (C2) to determine whether the GO Cluster A transcriptomic changes were recapitulated in vivo. To do so we focused on the response to stilbenoid, C21-steroid hormone metabolism, long-chain fatty acid transport, and positive regulation of angiogenesis (Figure 3a). We first examined the response to stilbenoid and the C21-steroid hormone metabolic process. The GO term “response to stilbenoid” encompasses diverse biological processes but includes key metabolic enzymes such as Cyp2a5, which stilbenoids including resveratrol regulate [14]. Cyp2a5 expression showed a transient decreasing trend at 12 h post-PH that did not reach statistical significance (*p* > 0.05) and then gradually recovered to baseline by 168 h (Figure 3b). Hsd3b5, which also belongs to this GO term and encodes a key steroid-degrading enzyme [15], was markedly downregulated at 6 and 12 h but returned to preoperative levels by 168 h (Figure 3c). Together, these findings indicate rapid suppression of constitutive steroid and xenobiotic catabolism immediately after liver resection.

We next examined angiogenic signaling activation. Expression of Vegfr1, a pivotal pro-angiogenic receptor, was significantly upregulated at 6 h post-PH (Figure 3d). To characterize this response further, we examined the subcellular localization of HIF-1α, the master transcriptional regulator of angiogenesis. Before PH, HIF-1α was predominantly cytoplasmic in non-parenchymal cells; at 6 and 12 h post-PH, however, it distinctly translocated to the nuclei of both hepatic sinusoidal endothelial cells (SECs) and hepatocytes (Figure 3e). This localization shift supports activation of angiogenic transcriptional programs in multiple hepatic cell types.

Finally, to assess long-chain fatty acid transport, we examined expression of CD36, a major fatty acid translocase [16]. By immunofluorescence, CD36 was virtually undetectable in hepatocytes before resection but was strongly induced at the hepatocyte plasma membrane 6–12 h post-PH; weak expression persisted through 336 h (Figure 3f).

Together, these findings validate the microarray results and show that coordinated metabolic reprogramming, acute lipid uptake, and rapid angiogenic signaling characterize the earliest phase of liver regeneration.

### 2.4. GO Cluster B: Dynamic Metabolic Shifts and ER Stress Response Following PH

**Figure 4:**
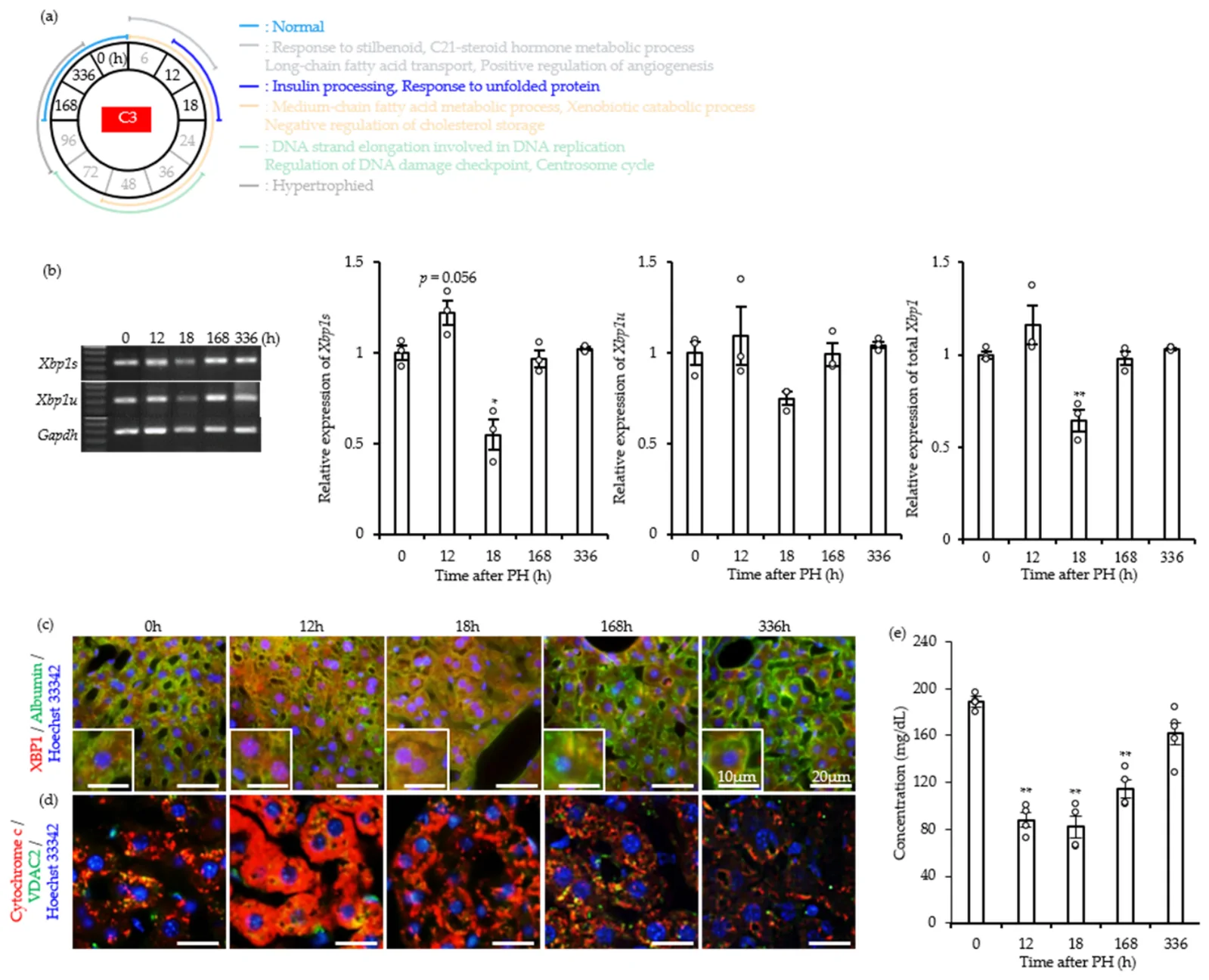
Insulin processing and endoplasmic reticulum (ER) stress in GO Cluster B. (**a**) Schematic overview of the post-PH time points and biological events validated in vivo. The circle center identifies the cluster analyzed, and highlighted text identifies the post-resection time points and corresponding GO terms examined. (**b**) Xbp1 expression. Total Xbp1 represents the combined expression of Xbp1u and Xbp1s. Values are means ± SEM, with individual data points shown (n = 3 mice per time point, empty circles). (**c**) Immunofluorescence staining of XBP1 (red), Albumin (green), and Hoechst 33342 (blue). Enlarged images appear in the lower left corner. Scale bars, 20 or 10 μm. (**d**) Immunofluorescence staining of cytochrome C (red), VDAC2 (green), and Hoechst 33342 (blue). Scale bar, 20 μm. (**e**) Serum glucose concentration. Values are means ± SEM, with individual data points shown (*n* = 4 or 5 mice per time point, empty circles). * *p* < 0.05, ** *p* < 0.01 vs. 0 h (one-way ANOVA followed by Dunnett’s post-hoc test).

As liver regeneration entered the mid-phase (12 and 18 h post-PH), transcriptomic profiling showed a shift in gene expression toward GO Cluster B (Sample Cluster 3; C3). Robust induction of genes associated with the response to unfolded proteins and insulin processing characterized this cluster (Figure 2d,e and Figure 4a), suggesting extensive proteostatic and metabolic remodeling in the remaining hepatocytes. We examined expression and processing of Xbp1, a canonical ER stress mediator, to validate the unfolded protein response (UPR) represented by GO Cluster B. RT-qPCR showed an increasing trend in the active spliced isoform Xbp1s at 12 h post-PH that approached statistical significance (*p* = 0.056; Figure 4b). By 18 h, however, Xbp1s expression declined significantly, whereas the unspliced isoform (Xbp1u) did not show a statistically significant change (*p* > 0.05), contributing to reduced overall XBP1 protein levels.

We next used immunofluorescence to characterize UPR activation through the subcellular localization of XBP1. At 0 h, XBP1 was confined predominantly to the cytoplasm of hepatocytes. At 12 and 18 h post-PH, by contrast, XBP1 showed pronounced nuclear translocation (Figure 4c), consistent with activity as a transcription factor during this stage. Cytochrome C and VDAC2 immunofluorescence further supported concurrent mitochondrial stress in regenerating hepatocytes. During the 12–18 h period, altered mitochondrial cytochrome C/VDAC2 staining patterns indicated transient organelle-level stress coinciding with the transcriptomic surge in GO Cluster B (Figure 4d).

We measured systemic blood glucose concentrations to examine the broader metabolic adaptation accompanying the insulin-processing signature in GO Cluster B [17]. Relative to the pre-resection baseline, circulating glucose decreased significantly from 24 to 168 h post-PH and returned to baseline by 336 h (Figure 4e). Together, these findings indicate that GO Cluster B activation reflects coordinated cellular stress and metabolic adaptation that precede and accompany the transition to the proliferative phase.

### 2.5. GO Cluster C: Metabolic Reprogramming and Systemic Lipid Dynamics Preceding Cell Proliferation

**Figure 5:**
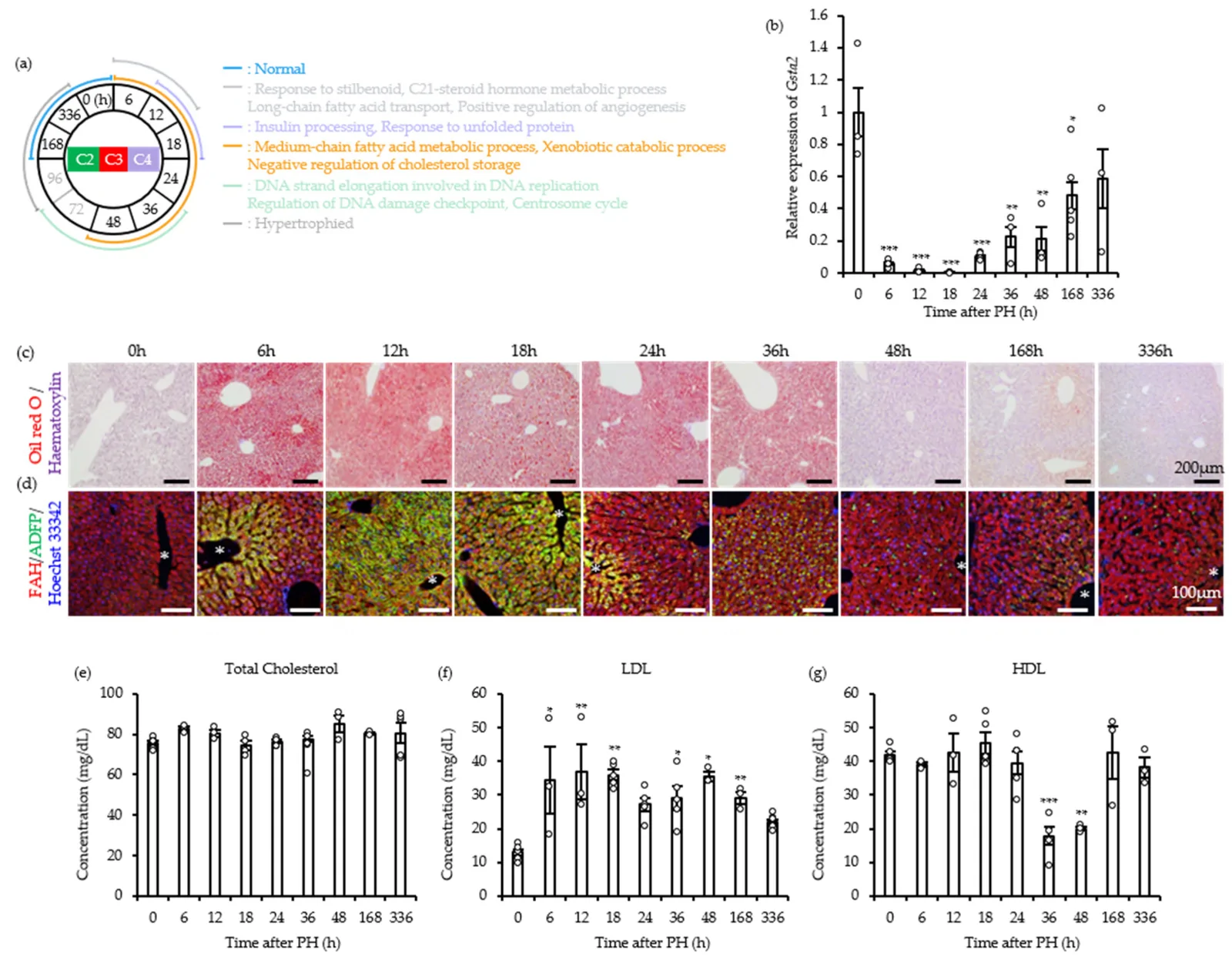
Xenobiotic catabolism, medium-chain fatty acid (MCFA) metabolism, and cholesterol storage in GO Cluster C. (**a**) Schematic overview of the post-PH time points and biological events validated in vivo. The circle center identifies the cluster analyzed, and highlighted text identifies the post-resection time points and corresponding GO terms examined. (**b**) *Gsta2* expression. Values are means ± SEM, with individual data points shown (*n* = 3–5 mice per time point, empty circles). * *p* < 0.05, ** *p* < 0.01, *** *p* < 0.001 vs. 0 h (one-way ANOVA followed by Dunnett’s post-hoc test). (**c**) Oil Red O staining with hematoxylin counterstaining. Scale bar, 200 μm. (**d**) Immunofluorescence staining of FAH (red), ADFP (green), and Hoechst 33342 (blue). Scale bar, 100 μm; *, central vein. (**e**–**g**) Serum total cholesterol (**e**), LDL (**f**), and HDL (**g**). Values are means ± SEM, with individual data points shown (*n* = 3 or 5 mice per time point, empty circles). * *p* < 0.05, ** *p* < 0.01, *** *p* < 0.001 vs. 0 h (one-way ANOVA followed by Dunnett’s post-hoc test).

Transcriptomic profiling identified GO Cluster C (Sample Clusters 3–5) as a major functional module. From 6 to 48 h post-PH, this module showed sustained downregulation of genes involved in medium-chain fatty acid metabolism, xenobiotic catabolism, and negative regulation of cholesterol storage (Figure 5a). This coordinated suppression suggests that hepatic priorities shift as metabolic resources are redirected from constitutive functions toward tissue regeneration. To validate this metabolic reprogramming in vivo, we measured expression of Gsta2, a key phase II drug-metabolism enzyme, as a marker of xenobiotic catabolism [18]. Expression of Gsta2 was significantly reduced from 6 to 168 h post-PH and returned to baseline only by 336 h (Figure 5b), confirming transient suppression of the liver’s detoxification capacity during the peak regenerative period.

We next examined lipid dynamics histologically to investigate medium-chain fatty acid metabolism. Oil Red O (ORO) staining revealed extensive lipid accumulation throughout the liver between 6 and 36 h post-PH (Figure 5c). Spatially, lipids initially accumulated in hepatocytes surrounding the central vein at 6 h, spread throughout the hepatic parenchyma at 12 and 18 h, and returned to a predominantly pericentral distribution by 24 h (Figure 5d). In adjacent tissue sections, the spatial distribution of ADFP (Perilipin 2) immunofluorescence closely overlapped that of lipid droplets (Figure 5d), providing molecular evidence of droplet localization and associated coat-protein expression in regenerating hepatocytes.

Finally, we profiled systemic lipoprotein dynamics to investigate negative regulation of cholesterol storage. Although total cholesterol did not change significantly (*p* > 0.05; Figure 5e), individual lipoprotein fractions showed more pronounced, divergent alterations. Except at 24 h, LDL concentrations were significantly elevated from 6 to 168 h post-PH (Figure 5f). By contrast, HDL concentrations decreased significantly at 36 and 48 h (Figure 5g) and reached their lowest levels as the liver entered the proliferative phase.

Together, these findings show extensive reprogramming of lipid metabolism during the pre-proliferative phase. Coordinated induction of ADFP, a major coat protein required for lipid-droplet stabilization, accompanied lipid accumulation. Together with systemic HDL depletion, these changes represent distinctive physiological hallmarks of the liver’s preparation for cell division.

### 2.6. GO Cluster D: Coordination of DNA Replication and Cell-Cycle Progression

**Figure 6:**
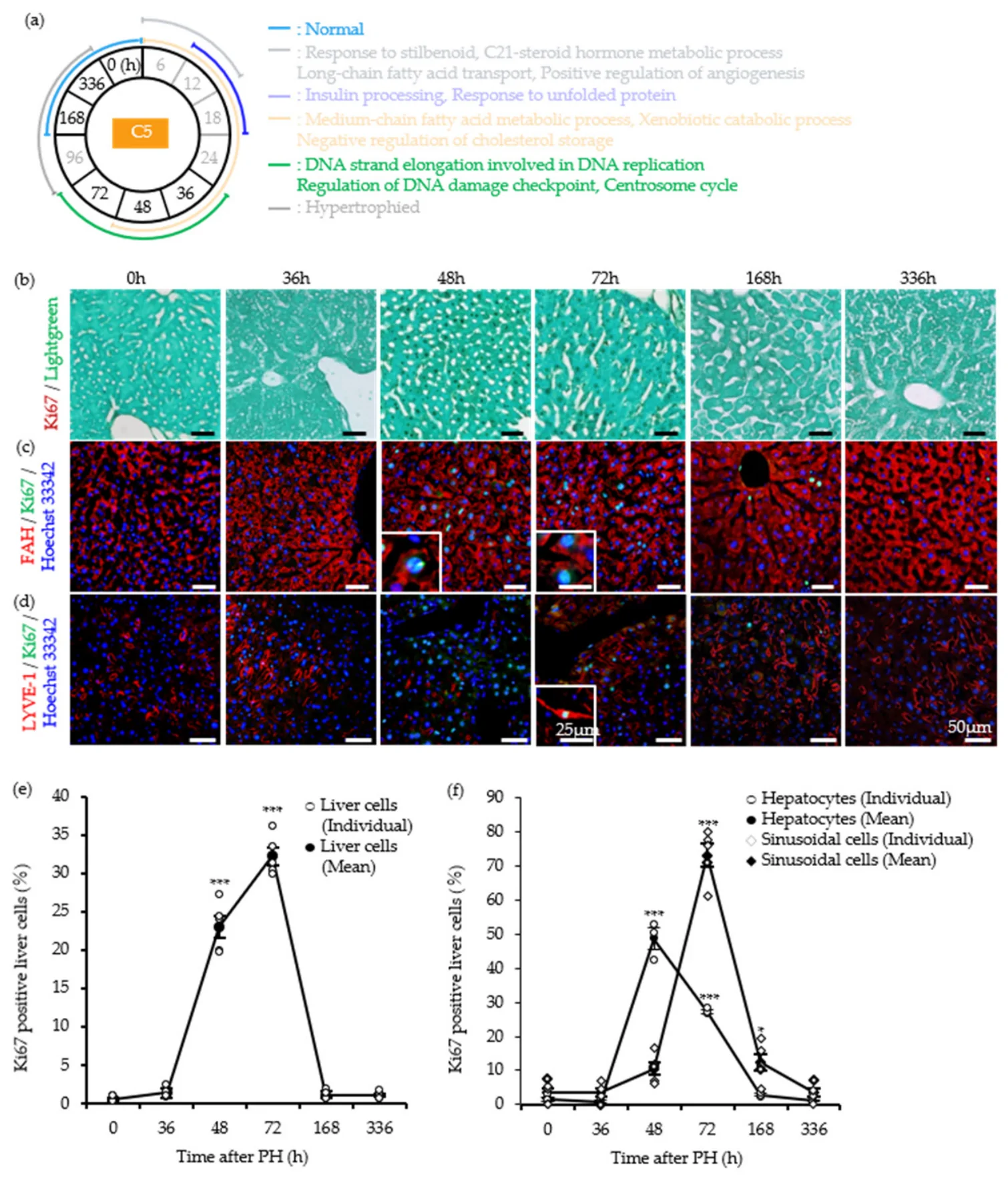
Cell-cycle progression in hepatocytes and sinusoidal endothelial cells in GO Cluster D. (**a**) Schematic overview of the post-PH time points and biological events validated in vivo. The circle center identifies the cluster analyzed, and highlighted text identifies the post-resection time points and corresponding GO terms examined. (**b**) Immunohistochemical staining of proliferating cells with an anti-Ki-67 antibody and light green counterstain. Scale bar, 50 μm. (**c**,**d**) Immunofluorescence staining of FAH (red, (**c**)), LYVE-1 (red, (**d**)), Ki-67 (green), and Hoechst 33342 (blue). Enlarged images appear in the lower left corner. Scale bars, 25 μm. (**e**) Ki-67-positive cells as a percentage of total liver cells. Solid lines and filled black symbols indicate means; open circles indicate individual data points (*n* = 5). Values are means ± SEM. (**f**) Ki-67-positive hepatocytes (● and ○) and sinusoidal endothelial cells (◆ and ◇), shown as percentages. Solid lines and filled black symbols indicate means; white symbols indicate individual data points (*n* = 3 or 5). Values are means ± SEM. * *p* < 0.05, *** *p* < 0.001 vs. 0 h (one-way ANOVA followed by Dunnett’s post-hoc test).

During the peak proliferative phase (36–72 h post-PH), the transcriptomic landscape shifted toward GO Cluster D (Sample Cluster 5). Functional enrichment analysis showed that genes involved in DNA strand elongation during replication, DNA damage-checkpoint regulation, and the centrosome cycle predominantly characterized this cluster (Figure 6a). These findings suggest coordinated activation of the cell-cycle machinery following the preceding phases of metabolic reprogramming and ER stress. We quantified cellular proliferation using Ki-67 as a marker of actively cycling cells to validate these transcriptomic signatures at the tissue level. The total number of proliferating cells in the liver increased significantly at 48 and 72 h post-PH (Figure 6b,e). Consistent with the GO Cluster D functional categories, the proliferative response followed distinct temporal patterns among hepatic cell populations. Hepatocyte proliferation increased significantly at both 48 and 72 h (Figure 6c,f). In contrast, significant proliferation of SECs was detected at 72 and 168 h post-PH (Figure 6d,f). Thus, hepatocyte proliferation preceded that of non-parenchymal cells, reflecting coordinated but cell type-specific re-entry into the cell cycle during liver regeneration.

Proliferation of both hepatocytes and SECs began immediately after the marked reduction in circulating HDL observed between 36 and 48 h. Together, these findings validate the microarray-derived identification of GO Cluster D and delineate a temporal sequence of physiological events during liver regeneration. The sequence links established adaptations, including ER stress and metabolic reprogramming, to characteristic fluctuations in circulating HDL and LDL concentrations that precede large-scale cellular proliferation.

## 3. Discussion

Our integrated microarray and histological analyses reproduced several established observations in PH models, including early angiogenic signaling [19], mitochondrial swelling [20,21], dynamic Xbp1 expression [22,23], and transient lipid accumulation [24]. Recapitulation of these hallmark events supports the ability of our bioinformatics-driven approach to capture the in vivo state of the regenerating liver. A notable feature of our transcriptomic analysis was the identification of Cluster 6 (96–168 h post-PH). Although GO analysis identified less distinct functional categories than in earlier clusters, enrichment of the cholesterol metabolic process was significant (nominal *p* = 0.046). This finding is consistent with the observation that serum LDL and blood glucose concentrations had not fully returned to baseline even at 168 h (Figure 4e and Figure 5f). These data suggest that subtle functional alterations persist one week after resection and that the liver has not fully returned to a stable pre-resection state, supporting designation of this period as a distinct physiological cluster.

To assess consistency with recent high-throughput sequencing studies, we compared our transcriptional dynamics with post-2019 RNA-seq and single-cell transcriptomic benchmarks of mouse liver regeneration [25,26]. We specifically examined the established regeneration-related markers *Il6*, *Stat3*, *Hgf*, *Tgfb1*, *Tgfb2*, and *Tgfb3*. Across distinct regenerative phases, the temporal microarray profiles were broadly consistent with recent RNA-seq findings (Figure S1). *Stat3* exhibited a rapid transcriptional induction during the early priming window (Cluster C2), echoing the immediate-early transcriptomic spikes described in recent single-cell chronoatlas datasets [26]. Among the three *Hgf* probe sets, one showed the highest-magnitude response (FC = 2.31) and peaked during the primary proliferative phase (Cluster C4), whereas the other two probe sets showed earlier induction, consistent with the time-resolved transcriptional changes reported in partial hepatectomy models [25]. Conversely, *Tgfb2* displayed transient downregulation during the early phase (Cluster C3), reflecting the temporary shutdown of anti-proliferative signals necessary to permit cell-cycle progression before returning toward late-stage termination regulation. Concordance across sequencing platforms therefore supports the consistency of our microarray dataset with previously reported expression patterns during hepatic regeneration.

Our findings provide additional insight into lipid regulation during liver regeneration. The observed decreasing trend in Cyp2a5 expression suggests possible suppression of the stilbenoid response. Because stilbenoids, including resveratrol, exert protective effects against hepatic lipid accumulation [24], this early suppression may weaken hepatocellular defense mechanisms and thereby contribute to the initiation of transient steatosis. This pathway remained significantly downregulated in C4 (24–48 h) but stabilized in C5 (36–72 h), coincident with resolution of lipid accumulation (Figure 5c). This pattern suggests that restoration of these protective pathways coincides with and may support metabolic recovery. The early regenerative phase was also characterized by increased CD36 expression in hepatocytes between 6 and 12 h (Figure 3f), indicative of enhanced long-chain fatty acid uptake. Under the systemic hypoglycemia observed here (Figure 4e), increased fatty acid uptake likely provides a compensatory energy source for the rapidly rising metabolic demands of regeneration [27].

A central observation was the marked reduction in circulating HDL at 36 and 48 h post-PH (Figure 5g). HDL is the principal carrier of cholesterol and phospholipids, which provide essential substrates for membrane synthesis during rapid cellular proliferation. Because inhibition of HDL synthesis has been shown to impair cytokinesis and promote multinucleation [28], the transient decrease in serum HDL may reflect a metabolic adaptation potentially associated with systemic lipid mobilization during liver regeneration. However, functional lipid flux and direct hepatic lipid uptake were not measured, and no causal relationship with hepatocyte proliferation was established. This interpretation therefore remains a working hypothesis requiring further investigation. Integrating these physiological events, we propose that early responses associated with hemodynamic alterations and shear stress [29] may initiate a sequence in which lipid metabolic reprogramming and proteostatic stress occur before the systemic proliferative response.

Beyond validating individual physiological cascades, our chronologically resolved, multi-replicate dataset revealed temporal uncoupling between macroscopic recovery and molecular-level (transcriptional) convergence. Near-complete transcriptomic normalization occurred well after the liver-to-body weight ratio recovered at 96 h (Figure 1b,c; see also Cluster 6 above). This discrepancy suggests that liver weight and serum biochemical parameters, which have traditionally been used to define completion of liver regeneration [1,2,3], reflect restoration of organ mass and gross physiological function but do not necessarily indicate that hepatocytes have returned to their pre-injury transcriptional and metabolic state. Macroscopic recovery therefore should not be equated with complete regeneration; molecular endpoints may assess completion of the regenerative program more comprehensively than weight or serum markers alone. At 336 h, one of the three biological replicates remained in Cluster 6, with a small number of genes still differentially expressed (12 DEGs; Figure 2a, Table S1). Notably, no GO terms were statistically enriched among these DEGs, suggesting that these residual transcriptional changes do not form a clearly coordinated pathway-level response at this late stage.

The residual changes may reflect both low-level functional or metabolic adjustment and stochastic or technical variation. Persistent alterations in specific regulatory genes may indicate continued metabolic adaptation as the liver returns to resting homeostasis. For example, Fgl1 is a liver-derived hepatokine involved in metabolic regulation, whereas Fabp5 is an intracellular lipid chaperone involved in fatty acid metabolism [30,31]. Thus, low-level functional and metabolic adjustment may continue after gross liver mass has been restored.

Another subset of the lingering DEGs may instead reflect residual biological or statistical noise. Major rodent acute-phase reactants, including Saa1, Saa2, and Apcs, are highly sensitive markers that readily fluctuate in response to minor physiological stress or inter-individual variations [32]. Moreover, persistent low-level expression of Saa transcripts characterizes the late resolution phase after an acute inflammatory response [33]. Although intriguing, this observation cannot establish whether the difference reflects genuine biological variation because the sample size was small (*n* = 3 per time point). At 336 h, transcriptomic divergence resulted from one animal remaining in Cluster 6 while the other two returned to baseline. This single-animal pattern cannot be distinguished definitively from technical noise or stochastic variation in cell-cycle exit. While subtle differences in surgical stress or individual recovery kinetics remain theoretical possibilities, our study was not designed to establish genuine biological heterogeneity. Future studies therefore require larger independent biological cohorts to determine rigorously whether genuine transcriptomic heterogeneity occurs during the late normalization phase of liver regeneration.

This study was limited to 10-week-old male mice. Because sex hormones influence liver regeneration and lipid metabolism [34], these findings may not fully represent regeneration in females. Studies including female mice are needed to identify potential sex-dependent differences in the molecular dynamics of liver regeneration. In addition, as reported in the ARRIVE checklist, group allocation was not formally randomized, and experimenters were not blinded during surgery or tissue sampling. Although objective bioinformatic and quantitative histological protocols were used to minimize bias, these methodological constraints should inform interpretation of the findings.

In summary, this study integrates previously fragmented observations into a continuous temporal framework of liver regeneration and demonstrates how a bioinformatics-driven approach can characterize the temporal complexity of in vivo biological processes.

## 4. Materials and Methods

### 4.1. Mice

All animal experiments followed the guidelines of the Nihon University Animal Care and Use Committee and the Nihon University Rules concerning Animal Care and Use. Male C57BL/6J mice (10 weeks old; Charles River, Sulzfeld, Germany) were used.

### 4.2. 70% PH

We performed 70% PH according to a previously described procedure [35]. Briefly, 10-week-old mice were anesthetized with isoflurane (FUJIFILM Wako Pure Chemical Corporation, Osaka, Japan). After restraining the animals, we made a 3-cm midline incision through the abdominal skin and muscle to expose the xiphoid process. We ligated the median and left lateral lobes near their base with 4-0 silk surgical thread and then resected them, removing approximately 70% of the total liver mass. The abdominal incision was then closed by suturing. At 0, 6, 12, 18, 24, 36, 48, 72, 96, 168, and 336 h after PH, mice were euthanized for analysis, and the remnant liver tissue was collected after exsanguination. At each time point, a primary cohort of three mice (*n* = 3) was used for initial transcriptomic profiling (microarray), serum biochemical assays, and liver-to-body weight ratio measurements. For RT-qPCR and quantitative histological analyses, we supplemented this primary cohort with additional independent biological replicates (*n* = 1–2) prepared under identical surgical, housing, and sampling conditions, yielding *n* = 3–5 mice per time point. In accordance with ARRIVE guidelines, mice were assigned to time-point groups without formal randomization, and neither surgery nor tissue harvesting was performed under blinded conditions.

### 4.3. Serum Glucose Concentration Quantification

We measured serum glucose concentrations with the LabAssay Glucose Kit (Wako Pure Chemical, Osaka, Japan) according to the manufacturer’s protocols.

### 4.4. Total Serum Cholesterol, HDL, and LDL Quantification

We measured serum total cholesterol, HDL, and LDL concentrations with the Cholesterol Assay Kit–HDL and LDL/VLDL (ab65390; Abcam, Cambridge, UK) according to the manufacturer’s protocols.

### 4.5. Histochemistry

We perfusion-fixed livers with 4% paraformaldehyde in PBS (Wako Pure Chemical) and embedded them in paraffin or Tissue-Tek O.C.T. compound (Sakura Finetek, Tokyo, Japan). For immunofluorescence staining, we washed frozen sections with TBS and blocked nonspecific antibody binding by incubating them for 1 h at room temperature in TBS containing 10% normal goat or horse serum (Vector Laboratories, Newark, CA, USA) and 1% bovine serum albumin (BSA; Sigma-Aldrich, St. Louis, MO, USA). We then incubated the sections with primary antibodies according to standard procedures. Primary antibodies included rabbit anti-HIF-1α polyclonal antibody (1:100 dilution; PA3-16521; Invitrogen, Carlsbad, CA, USA), rabbit anti-cytochrome C monoclonal antibody (1:100 dilution; ab133504; Abcam), rabbit anti-XBP1 polyclonal antibody (1:50 dilution; ab37152; Abcam), rabbit anti-fumarylacetoacetate hydrolase polyclonal antibody (1:200 dilution; ab81087; Abcam), goat anti-VDAC2 polyclonal antibody (1:50 dilution; ab37985; Abcam), rat anti-Ki67 monoclonal antibody (1:200 dilution; 11-5698; eBioscience, San Diego, CA, USA), rat anti-CD36 monoclonal antibody (1:50 dilution; ab80080; Abcam), rat anti-LYVE-1 monoclonal antibody (1:400 dilution; 14-0443; eBioscience, Inc., San Diego, CA, USA), and chicken anti-ADFP polyclonal antibody (1:400 dilution; ab37516; Abcam). For secondary detection of immune complexes, we used Alexa Fluor 488 donkey anti-goat IgG (1:2000 dilution), Alexa Fluor 488 goat anti-chicken IgG and goat anti-rat IgG (1:2000 dilution), and Alexa Fluor 594 goat anti-rat IgG and goat anti-rabbit IgG (1:2000 dilution). All antibodies were diluted in TBS containing 1% BSA. Cell nuclei were counterstained with Hoechst 33342 (5 μg/mL) in TBS.

For Oil Red O staining, we washed frozen sections with TBS and incubated them in 60% isopropyl alcohol for 1 min at room temperature. We then stained the sections for 15 min at 37 °C with filtered Oil Red O solution (0.5 g in 100 mL isopropyl alcohol), washed them sequentially with 60% isopropyl alcohol and distilled water, and counterstained them with Mayer’s hematoxylin solution (Wako Pure Chemical). Finally, the sections were mounted with Fluoromount G.

For immunohistochemical analysis, we deparaffinized the paraffin sections, washed them with distilled water, and stained them with a Vectastain ABC kit (Vector Laboratories). The primary antibody used was a rat anti-Ki67 monoclonal antibody (1:200 dilution). Sections were counterstained with Light Green. For each animal, we captured five randomly selected, non-overlapping fields per tissue section at ×200 magnification for quantitative Ki-67 analysis (*n* = 5 fields per mouse). Ki-67-positive hepatocytes were quantified using ImageJ software (version 1.53e). Image acquisition and counting were not blinded to time point, as disclosed in the study design; however, we applied standardized thresholds and objective counting parameters consistently across all samples.

### 4.6. RNA Extraction

We extracted total RNA with TRIzol reagent (Invitrogen) and an RNeasy Mini Kit (Qiagen, Hilden, Germany) according to the manufacturer’s instructions. We assessed RNA quality with a NanoDrop^®^ ND-1000 UV–visible spectrophotometer (NanoDrop Technologies, Wilmington, DE, USA). After DNase I digestion, we reverse-transcribed 500 ng of total RNA with SuperScript^®^ VILO™ Master Mix (Invitrogen).

### 4.7. RT (Reverse Transcription)–PCR

We used reverse-transcribed cDNA as the template for gene-expression analysis with 10× Ex Taq Buffer, dNTP Mixture, and Ex Taq (Takara Bio Inc., Kusatsu, Japan) according to the manufacturer’s instructions. The following primer sets were used to detect ER stress markers: Xbp1u (X-box binding protein 1 isoform Xbp1(u)), sense, 5′-GCAGGTGCAGGCCCAGTTGTCAC-3′ and antisense, 5′-CCCCACTGACAGAGAAAGGGAGG-3′; Xbp1s (X-box binding protein 1 isoform Xbp1(s)), sense, 5′-GGGTCTGCTGAGTCCGCAGCAG-3′ and antisense, 5′-CCCCACTGACAGAGAAAGGGAGG-3′. The primer set for Gapdh (glyceraldehyde-3-phosphate dehydrogenase) was used as the internal reference (sense, 5′-GGGAAGCTTGTCATCAATGG-3′ and antisense, 5′-GTTGTCATGGATGACCTTGG-3′). To prevent genomic DNA amplification, all primer sets spanned exon-exon junctions. PCR conditions comprised initial denaturation for 2 min at 95 °C; 30 cycles of 30 s at 96 °C, 30 s at 60 °C, and 60 s at 72 °C; and final extension for 5 min at 72 °C. Each sample was analyzed in triplicate. We separated PCR products on agarose gels and visualized them with DBA stain (Bioatlas, Tartu, Estonia).

### 4.8. Real Time-PCR

We quantified gene-expression with TaqMan Fast Universal PCR Master Mix (Applied Biosystems, Foster City, CA, USA) according to the manufacturer’s instructions. TaqMan Gene Expression Assay probes (Applied Biosystems) were used to quantify Vegfr1 (Mm00438980_m1), Cyp2a5 (Mm00487248_g1), Gsta2 (Mm00451269_m1), and Hsd3b5 (Mm00440939_m1). We used a mouse Gapdh TaqMan probe (glyceraldehyde-3-phosphate dehydrogenase; GenBank accession no. NM_008084.2; 4352339E, Applied Biosystems) as the endogenous control. We subjected RT products (2 μL) to quantitative PCR in a final volume of 10 μL with TaqMan Fast Universal PCR Master Mix (Applied Biosystems) on an ABI 7500 thermocycler. Each sample was assayed in triplicate (*n* = 5 biological replicates per time point).

### 4.9. Microarray Data Analysis

We extracted total RNA from liver tissue collected before and after PH with TRIzol reagent (Invitrogen) and the RNeasy Mini Kit (Qiagen) according to the manufacturer’s instructions. Sample preparation and microarray processing were performed by Filgen, Inc. (Nagoya, Japan). We analyzed microarray data in R (version 4.0.3; http://www.r-project.org/ (accessed on 2 October 2020)) with packages from the Bioconductor project (http://www.bioconductor.org/ (accessed on 2 October 2020)). Raw data were summarized and normalized using the MAS5.0 algorithm implemented in the Bioconductor package affy (http://www.bioconductor.org/packages/2.0/bioc/html/affy.html (accessed on 2 October 2020)) [36]. We selected MAS5.0 instead of multi-sample normalization algorithms such as RMA because it processes each array independently and was considered suitable for this extensive time-course dataset. This approach was intended to preserve the dynamic range of expression measurements, reduce potential fold-change compression, and facilitate detection of temporal expression changes across the 11 time points. Microarray samples were clustered using the X-means method according to Ishioka et al. [37]. Three-dimensional principal component analysis (3D-PCA) was performed using Spotfire DecisionSite for Functional Genomics (TIBCO Software Inc., Palo Alto, CA, USA). Differentially expressed genes were identified across the post-PH time points. Specifically, DEGs were defined as genes with an absolute log_2_ fold-change > 1.0 and an FDR-adjusted *p*-value < 0.05 relative to the 0 h control group. Lists of upregulated and downregulated genes were generated using the Functional Annotation Chart Tool in DAVID Bioinformatics Resources (https://davidbioinformatics.nih.gov/, accessed on 1 October 2026). GO term clustering was performed in Spotfire using the diagram function. REVIGO treemaps were generated using the rrvgo package with *p*-values, fold enrichment, and gene counts as input scores.

### 4.10. Statistical Analyses

Data are presented as means ± s.e.m. All statistical tests were two-sided, with α = 0.05. Statistical analyses were performed using one-way analysis of variance (ANOVA). We used Tukey’s post-hoc test for comparisons among multiple groups and Dunnett’s post-hoc test to compare post-PH time points with the 0 h control group. Because of the sample size per group, we did not formally evaluate statistical assumptions such as normality and homoscedasticity; however, we visually inspected data distributions and variances before parametric testing. A *p*-value < 0.05 was considered statistically significant.

## 5. Conclusions

Taken together, this integrated analysis provides a refined temporal framework for liver regeneration and shows that the sequence of metabolic and tissue remodeling extends beyond macroscopic recovery.

## Data Availability

All microarray datasets generated or analyzed in this and previous studies [13] are available from the NCBI GEO repository under accession GSE278618.

## Supplementary Materials

The following supporting information can be downloaded at: https://www.mdpi.com/article/10.3390/ijms27198829/s1.

## Abbreviations

The following abbreviations are used in this manuscript:

PH	Partial hepatectomy
DEGs	Differentially expressed genes
GO	Gene ontology
PCA	Principal component Analysis
