# Supplementary material for: Elucidating Temporally Ordered Physiological Events During Liver Regeneration via Integrated Transcriptomic-Histological Profiling
Source: Int J Mol Sci. 2026 Oct 2;27(19):8829. doi: 10.3390/ijms27198829 (PMC13665515; doi:10.3390/ijms27198829)
Supplement: Supplementary file 1 [file ijms-27-08829-s001.zip › Figure S1.pdf]

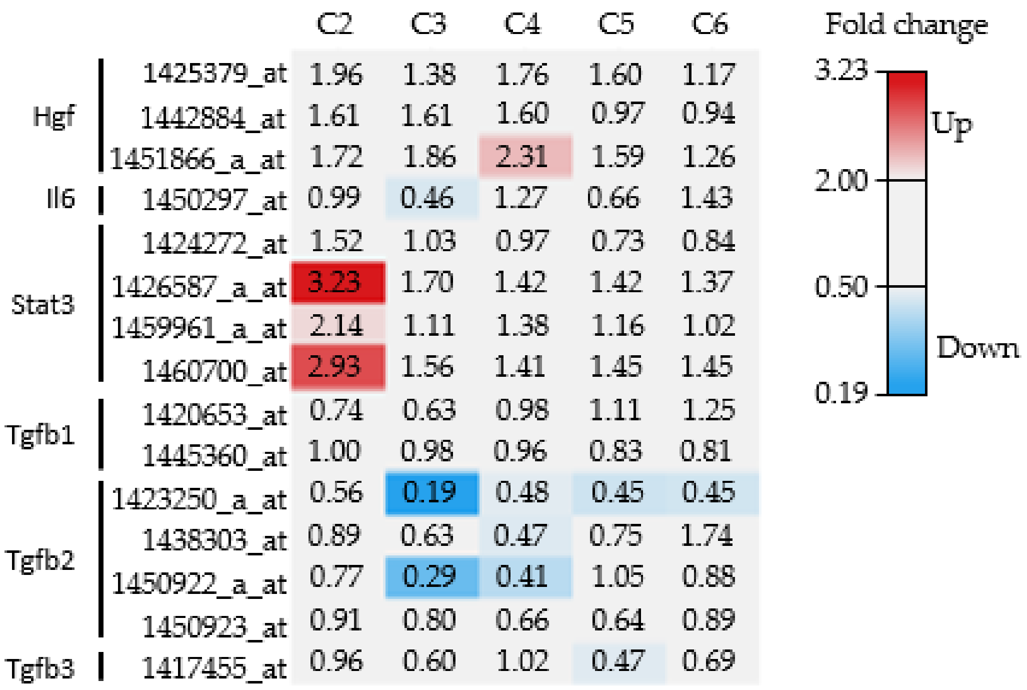

Figure S1. Temporal expression profiles of regeneration-related genes. The heatmap shows the relative expression profiles of representative regeneration-related genes (*Il6*, *Stat3*, *Hgf*, *Tgfb1*, *Tgfb2*, and *Tgfb3*) across temporal clusters C2–C6. Affymetrix Probe Set IDs and the corresponding gene symbols are shown in the row labels. Values within the cells indicate fold changes relative to Cluster 1 (C1). Red and blue shading denote upregulation and downregulation, respectively, relative to C1.
