# Supplementary material for: Elucidating Temporally Ordered Physiological Events During Liver Regeneration via Integrated Transcriptomic-Histological Profiling
Source: Int J Mol Sci. 2026 Oct 2;27(19):8829. doi: 10.3390/ijms27198829 (PMC13665515; doi:10.3390/ijms27198829)
Supplement: Supplementary file 1 [file ijms-27-08829-s001.zip › Table S1.pdf]

Probe ID	Gene Symbol	Gene Name / Description	Biological Function (NCBI / UniProt Annotation)
1452445_at	*Slc41a2*	Solute carrier family 41 member 2	Transports magnesium ions across the plasma membrane and maintains cellular cation homeostasis.
1449326_x_at	*Saa2*	Serum amyloid A 2	Functions as a major acute-phase reactant involved in the systemic inflammatory response and lipid transport.
1419075_s_at, 1450788_at	*Saa1*	Serum amyloid A 1	Functions as a major acute-phase protein induced during liver injury and the resolution of inflammation.
1449824_at	*Prg4*	Proteoglycan 4	Acts as an extracellular matrix proteoglycan that regulates cell-matrix interactions and protects tissues.
1427747_a_at	*Lcn2*	Lipocalin 2	Acts as an acute-phase responder that mediates iron trafficking, cellular survival, and hepatoprotection.
1424599_at	*Fgl1*	Fibrinogen-like 1	Functions as a liver-derived hepatokine that regulates hepatocyte proliferation and metabolic homeostasis.
1416021_a_at, 1416022_at	*Fabp5*	Fatty acid binding protein 5	Functions as an intracellular lipid chaperone that facilitates fatty acid transport and lipid metabolism.
1417339_a_at	*Dynll1*	Dynein light chain LC8-type 1	Functions as a cytoskeletal motor component that regulates intracellular transport and apoptotic signaling.
1417441_at	*Dnajc12*	DnaJ heat shock protein member C12	Functions as a molecular chaperone involved in protein folding, quality control, and recovery from stress.
1419059_at	*Apcs*	Amyloid P component, serum	Functions as a major murine acute-phase protein that participates in innate immunity and matrix turnover.

Table S1. Comprehensive list and biological annotations of the 12 lingering differentially expressed genes (DEGs) in Cluster 6. The table provides the probe IDs, official gene symbols, full gene names, and detailed biological functions of all 12 DEGs (representing 10 unique genes) that remained significantly altered in Cluster 6. Biological functions were annotated using the NCBI, UniProt, and Gene Ontology databases and relevant published literature.
